# Supplementary material for: CellTypeAI: cell annotation for scRNA-seq using local generative-AI
Source: Bioinformatics. 2026 Aug 21;42(Suppl 2):btag425. doi: 10.1093/bioinformatics/btag425 (PMC13501282; doi:10.1093/bioinformatics/btag425)
Supplement: Supplementary file 1 [file btag425_supplementary_data.pdf]

Supplementary Table 1. Patient data table for Stroke PBMC dataset.		
**Study_ID**	4133-066	4133-079
**gender_usuk_label**	Male	Male
**Age_years**	60	72
**Ethnicity**	White	White
**NIHSS**	7	9
**Date_of_Enrollment**	44665	44706
**Time_of_symptom_onset**	44663.41667	44705.91667
**Time_symptoms_were_recognized**	NA	NA
**Time_last_known_to_be_neurologically_normal_(LSN/LKW)**	NA	NA
**Date/Time_Blood_Drawn_**	44665.40972	44706.42708
**blooddrawdiff**	47.833333	12.25
**Lesion_Location**	Left	Left
**Lacunar**	No	Unknown
**IV_Thrombolysis**	No	Yes
**Was_intra-arterial_thrombectomy_undertaken__?**	No	No
**stroke_AMPM**	AM	PM
**stroke_time_of_day**	2	4
**Is_ECG_available?_**	Yes	Yes
**Left_Ventricular_Hypertrophy?**	No	No
**Atrial_Fibrillation_(Afib)?**	No	No

Supplementary Methods 1: Stroke PBMC Processing

As part of the Stroke-Immune Pathways and Cognitive Trajectory (Stroke-IMPaCT) Study, venous blood was drawn from ischaemic stroke patients within 96 h of stroke symptom onset or time last seen well, as well as at the 6-9-month follow-up timepoint. Blood was collected in ethylenediaminetetraacetic acid (EDTA) tubes and processed within 4 h.

To obtain peripheral blood mononuclear cells (PBMCs), EDTA blood was diluted 1:1 with phosphate-buffered saline (PBS) supplemented with 2% foetal bovine serum (FBS) and centrifuged at 1200 g for 15 min at 20°C in SepMate™ PBMC Isolation Tubes (StemCell Technologies, Vancouver, Canada) with Ficoll-Paque™ PLUS density gradient media (Cat. No. 17144003; Cytiva Life Sciences, Amersham, UK). Following centrifugation, the top supernatant layer containing PBMCs was transferred to 50 mL falcon tubes, diluted with 20 mL PBS + 2% FBS and centrifuged at 500 g for 10 min at 20°C. Supernatant was discarded, and the cell pellet was resuspended in ACK Lysing Buffer (Cat. No. A1049201; Gibco, Waltham, USA) for 5 min at room temperature. PBS (10 mL) + 2% FBS was added and tubes centrifuged at 500 g for 5 min at 20°C. The cell pellet was resuspended in 20 mL FBS + 2% FBS and cells were counted before centrifugation at 500 g for 10 min at 20°C. Cells were resuspended at a final density of 5-6 × 106 cells / 500 µL CryoStor® CS10 Freezing Medium (Cat. No. 100-1061; StemCell Technologies), and frozen at -80°C in CoolCell™ Cell Freezing Vial Containers (Cat. No. 15542771; Corning, New York, USA). Finally, PBMC aliquots were transferred to liquid nitrogen until ready for use.

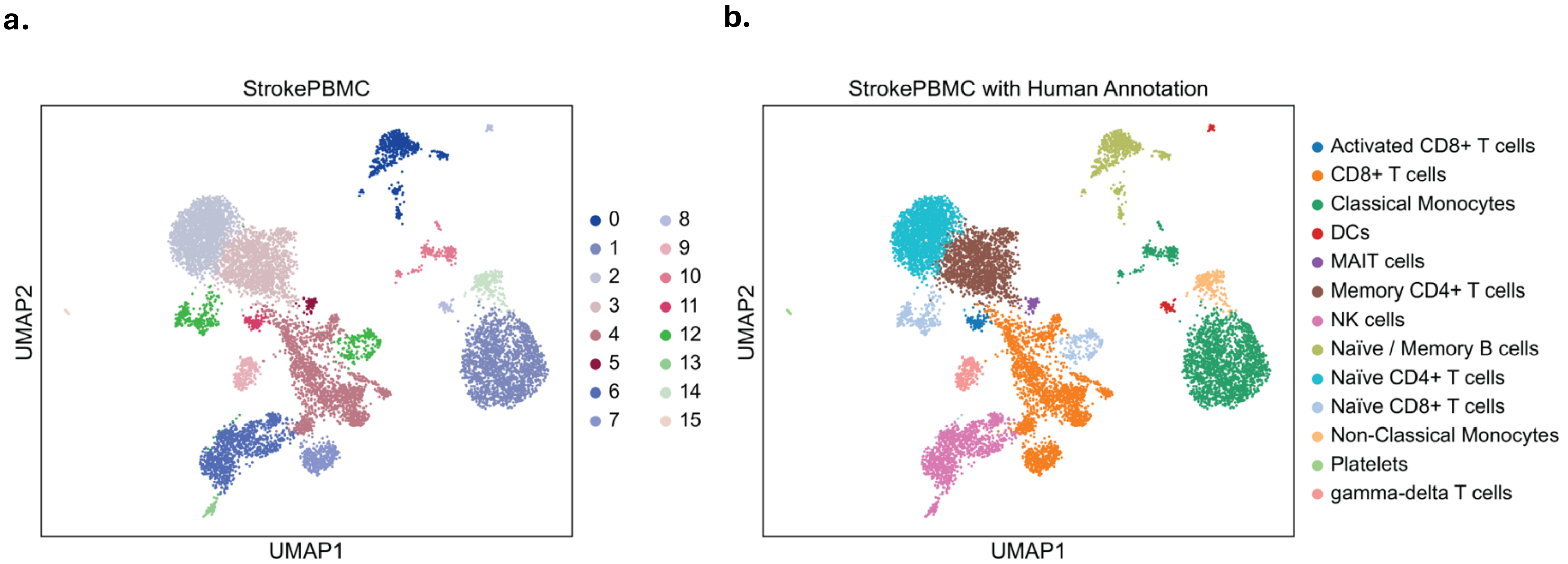

Supplementary Figure 1. UMAP visualization of Stroke PBMC dataset. a, UMAP visualization of leiden (0.3 resolution) clustering of Stroke PBMC single cell RNA sequencing samples. **b**, UMAP visualization of Stroke PBMC dataset with consensus human annotation of clusters depicted in **a**.

Supplementary Table 2. Contents of cell_context.json in tabular form, listing all tissues, cell types, and their defining marker genes.

Tissue	Cell Type	Markers
Immune system	Pro-B cells	CD19,CD79A,MS4A1,VPREB1,IGLL1,DNTT,RAG1,RAG2,CD24,KIT,IL7R
Immune system	Pre-B cells	CD19,CD79A,MS4A1,VPREB3,IGLL1,CD24,IL7R,PAX5,EBF1
Immune system	Naive B cells	CD19,MS4A1,CD79A,IGHM,IGHD,CD27,SELL,IL4R,CCR7,BACH2
Immune system	Memory B cells	CD19,MS4A1,CD79A,CD27,CD38,SDC1,IGHG1,IGHG2,IGHG3,IGHG4,IGHA1,IGHA2,AICDA,BANK1,PAX5
Immune system	Plasma B cells	SDC1,CD38,XBP1,PRDM1,SLAMF7,DERL3,MZB1,IGHG1,IGHG2,IGHG3,IGHG4,IGHA1,IGHA2,IGLC2,IGKC
Immune system	Naive CD8+ T cells	CD3D,CD3E,CD3G,CD8A,CD8B,CCR7,SELL,LEF1,TCF7,IL7R,CD27,CD45RA
Immune system	Naive CD4+ T cells	CD3D,CD3E,CD3G,CD4,CCR7,SELL,LEF1,TCF7,IL7R,CD27,CD45RA,FOXP1
Immune system	Memory CD8+ T cells	CD3D,CD3E,CD3G,CD8A,CD8B,CD44,IL7R,CD69,EOMES,TBX21,IFNG,GZMK,GZMB,CD45RO
Immune system	Memory CD4+ T cells	CD3D,CD3E,CD3G,CD4,CD44,IL7R,CD69,ICOS,CCR5,CCR6,CXCR3,TBX21,GATA3,RORC,FOXP3,CD45RO
Immune system	Effector CD8+ T cells	CD3D,CD3E,CD3G,CD8A,CD8B,GZMB,PRF1,IFNG,KLRG1,CX3CR1,TBX21,EOMES,CCL5,CD27,SELL
Immune system	Effector CD4+ T cells	CD3D,CD3E,CD3G,CD4,IFNG,TNF,IL2,IL4,IL17A,IL21,ICOS,CD40LG,TBX21,GATA3,RORC,BCL6,PRDM1
Immune system	CD4+ T regulatory cells	FOXP3,IL2RA,CTLA4,IKZF2,TNFRSF18,CD4,PTPRC,LAG3
Immune system	γδ-T cells	CD3D,CD3E,CD3G,TRDC,TRGC1,TRGC2,NKG7,KLRB1,KLRG1,CCL5,CD160
Immune system	MAIT cells	PTPRC,CD3D,SLC4A10,KLRB1,TRAV1-2,IL18R1,CCR6,RORC,ZBTB16
Immune system	Follicular Helper T cells	PTPRC,CD4,BCL6,CXCR5,PDCD1,ICOS,SLAMF6,CD40LG,MAF,ASCL2
Immune system	Invariant NKT cells	PTPRC,CD3D,ZBTB16,TRAV10,TRBV25,SLAMF1,SLAMF6,CD4,CD8A
Immune system	CD8+ NKT-like cells	CD3D,CD8A,CD8B,NCAM1,NKG7,KLRB1,KLRD1,GZMB,PRF1,TBX21,EOMES,SLAMF7
Immune system	CD4+ NKT-like cells	CD3D,CD4,NCAM1,NKG7,KLRB1,GZMK,TBX21,EOMES,SLAMF6
Immune system	Natural killer cells	NCAM1,NKG7,KLRF1,KLRD1,KLRB1,KLRC1,KLRC2,FCGR3A,GZMB,PRF1,EOMES,TBX21
Immune system	Innate Lymphoid Cell 1	PTPRC,ID2,IL7R,TBX21,IFNG,NKG7,NCR1,EOMES
Immune system	Innate Lymphoid Cell 2	PTPRC,ID2,IL7R,GATA3,IL5,IL13,IL1RL1,PTGDR2,ARG1
Immune system	Innate Lymphoid Cell 3	PTPRC,ID2,IL7R,RORC,IL22,IL17A,KIT,CCR6,NCR1
Immune system	Classical Monocytes	CD14,FCGR3A,CCR2,S100A8,S100A9,LYZ,VCAN,FCN1,CD33
Immune system	Non-classical monocytes	FCGR3A,CD14,CX3CR1,CSF1R,ITGAL,LST1,MS4A7,SIGLEC10,TCF7L2
Immune system	Intermediate monocytes	CD14,FCGR3A,CCR2,HLA-DRA,CD86,CCR5,ITGAX,CLEC10A
Immune system	Macrophages	CD68,CD163,MRC1,CSF1R,CD14,ITGAM,ADGRE1,APOE,C1QA,C1QB,C1QC,MARCO,MSR1
Immune system	Myeloid Dendritic cells	ITGAX,HLA-DRA,HLA-DRB1,HLA-DQA1,CD1C,FCER1A,CLEC10A,CD83,CD86,CCR7,IRF4,IRF8,SIRPA,CLEC9A
Immune system	Plasmacytoid Dendritic cells	IL3RA,CLEC4C,NRP1,TCF4,IRF7,IRF8,LILRA4,GZMB,SPIB,CD123
Immune system	cDC1	PTPRC,HLA-DRA,ITGAX,CLEC9A,XCR1,BATF3,IRF8,ID2,THBD
Immune system	cDC2	PTPRC,HLA-DRA,ITGAX,CD1C,SIRPA,CLEC10A,FCER1A,IRF4,NOTCH2,RELB
Immune system	Neutrophils	FCGR3B,CSF3R,MPO,ELANE,PRTN3,S100A8,S100A9,ITGAM,CEACAM8,CXCR1,CXCR2
Immune system	Eosinophils	CCR3,IL5RA,SIGLEC8,EPX,RNASE2,RNASE3,PRG2,CLC,HPGDS,PTGDR2
Immune system	Basophils	FCER1A,CD200R3,ENPP3,IL3RA,CD33,TPSAB1,TPSB2,HDC,GATA2
Immune system	Mast cells	KIT,FCER1A,CPA3,TPSAB1,TPSB2,MS4A2,ENPP3,CD63,CD9
Immune system	Granulocytes	MPO,ELANE,PRTN3,AZU1,S100A8,S100A9,CEACAM8,FCGR3B,CSF3R
Immune system	Platelets	ITGA2B,ITGB3,GP1BA,GP9,PPBP,PF4,SELP,TUBB1,CD9
Immune system	Megakaryocyte	ITGA2B,ITGB3,GP1BA,GP9,PF4,PPBP,VWF,MPL,FLI1,GATA1,TUBB1
Immune system	Erythroid-like and erythroid precursor cells	HBB,HBA1,HBA2,HBG1,HBG2,GYPA,GYPB,ALAS2,FECH,SLC4A1,EPOR,GATA1,KLF1
Immune system	HSC/MPP cells	CD34,KIT,THY1,SPINK2,AVP,PROM1,HOXA9,MEIS1,GATA2,MLLT3,CD90
Immune system	Progenitor cells	CD34,KIT,FLT3,MPO,AZU1,PRTN3,ELANE,GATA1,GATA2,MYB
Immune system	Endothelial	PECAM1,CDH5,VWF,ENG,ICAM1,SELE,SELP,CLDN5
Immune system	ISG expressing immune cells	ISG15,MX1,MX2,IFIT1,IFIT2,IFIT3,IFI6,IFI44L,OAS1,OAS2,OAS3,RSAD2,STAT1
Blood	Naive B cells	CD19,MS4A1,CD79A,IGHM,IGHD,CD27,SELL,IL4R,CCR7,BACH2
Blood	Memory B cells	CD19,MS4A1,CD79A,CD27,CD38,SDC1,IGHG1,IGHG2,IGHG3,IGHG4,IGHA1,IGHA2,AICDA,BANK1,PAX5
Blood	Plasma B cells	SDC1,CD38,XBP1,PRDM1,SLAMF7,DERL3,MZB1,IGHG1,IGHG2,IGHG3,IGHG4,IGHA1,IGHA2,IGLC2,IGKC
Blood	Naive CD8+ T cells	CD3D,CD3E,CD3G,CD8A,CD8B,CCR7,SELL,LEF1,TCF7,IL7R,CD27,CD45RA
Blood	Naive CD4+ T cells	CD3D,CD3E,CD3G,CD4,CCR7,SELL,LEF1,TCF7,IL7R,CD27,CD45RA,FOXP1
Blood	Memory CD8+ T cells	CD3D,CD3E,CD3G,CD8A,CD8B,CD44,IL7R,CD69,EOMES,TBX21,IFNG,GZMK,GZMB,CD45RO
Blood	Memory CD4+ T cells	CD3D,CD3E,CD3G,CD4,CD44,IL7R,CD69,ICOS,CCR5,CCR6,CXCR3,TBX21,GATA3,RORC,FOXP3,CD45RO
Blood	Effector CD8+ T cells	CD3D,CD3E,CD3G,CD8A,CD8B,GZMB,PRF1,IFNG,KLRG1,CX3CR1,TBX21,EOMES,CCL5,CD27,SELL
Blood	Effector CD4+ T cells	CD3D,CD3E,CD3G,CD4,IFNG,TNF,IL2,IL4,IL17A,IL21,ICOS,CD40LG,TBX21,GATA3,RORC,BCL6,PRDM1
Blood	T regulatory cells	FOXP3,IL2RA,CTLA4,IKZF2,TNFRSF18,CD4,PTPRC,LAG3
Blood	γδ-T cells	CD3D,CD3E,CD3G,TRDC,TRGC1,TRGC2,NKG7,KLRB1,KLRG1,CCL5,CD160
Blood	MAIT cells	PTPRC,CD3D,SLC4A10,KLRB1,TRAV1-2,IL18R1,CCR6,RORC,ZBTB16
Blood	Invariant NKT cells	PTPRC,CD3D,ZBTB16,TRAV10,TRBV25,SLAMF1,SLAMF6,CD4,CD8A
Blood	CD8+ NKT-like cells	CD3D,CD8A,CD8B,NCAM1,NKG7,KLRB1,KLRD1,GZMB,PRF1,TBX21,EOMES,SLAMF7
Blood	CD4+ NKT-like cells	CD3D,CD4,NCAM1,NKG7,KLRB1,GZMK,TBX21,EOMES,SLAMF6
Blood	Natural killer cells	NCAM1,NKG7,KLRF1,KLRD1,KLRB1,KLRC1,KLRC2,FCGR3A,GZMB,PRF1,EOMES,TBX21
Blood	Eosinophils	CCR3,IL5RA,SIGLEC8,EPX,RNASE2,RNASE3,PRG2,CLC,HPGDS,PTGDR2
Blood	Neutrophils	FCGR3B,CSF3R,MPO,ELANE,PRTN3,S100A8,S100A9,ITGAM,CEACAM8,CXCR1,CXCR2,SELL
Blood	Basophils	FCER1A,CD200R3,ENPP3,IL3RA,CD33,TPSAB1,TPSB2,HDC,GATA2
Blood	Classical Monocytes	CD14,FCGR3A,CCR2,S100A8,S100A9,LYZ,VCAN,FCN1,CD33,SELL
Blood	Non-classical monocytes	FCGR3A,CD14,CX3CR1,CSF1R,ITGAL,LST1,MS4A7,SIGLEC10,TCF7L2,ITGAM
Blood	Intermediate monocytes	CD14,FCGR3A,CCR2,HLA-DRA,CD86,CCR5,ITGAX,CLEC10A
Blood	cDC1	PTPRC,HLA-DRA,ITGAX,CLEC9A,XCR1,BATF3,IRF8,ID2,THBD
Blood	cDC2	PTPRC,HLA-DRA,ITGAX,CD1C,SIRPA,CLEC10A,FCER1A,IRF4,NOTCH2,RELB
Blood	Plasmacytoid Dendritic cells	IL3RA,CLEC4C,NRP1,TCF4,IRF7,IRF8,LILRA4,GZMB,SPIB,CD123,HLA-DRA
Blood	HSC/MPP cells	CD34,KIT,THY1,SPINK2,AVP,PROM1,HOXA9,MEIS1,GATA2,MLLT3,CD90
Blood	Progenitor cells	CD34,KIT,FLT3,MPO,AZU1,PRTN3,ELANE,GATA1,GATA2,MYB
Blood	Erythrocytes	HBB,HBA1,HBA2,GYPA,GYPB,SLC4A1,ALAS2,FECH,EPB41,SPTA1,CA1
Blood	Reticulocytes	HBB,HBA1,HBA2,GYPA,TFRC,ALAS2,RPS27,RPS19,RPL3,FTL,SLC4A1
Blood	Platelets	ITGA2B,ITGB3,GP1BA,GP9,PPBP,PF4,SELP,TUBB1,CD9,CLU
Liver	Hepatocytes	ALB,ASGR1,CPS1,HNF4A
Liver	Cholangiocytes	KRT19,KRT7,EPCAM,SOX9,CFTR,CLDN4,AQP1,SLC10A2,NR1H4
Liver	Hepatoblasts	AFP,EPCAM,KRT19,KRT8,DLK1,GPC3,ALB
Liver	Hepatic Stellate Cells	LRAT,RBP1,RELN,PDGFRB,DES,NGFR,GFAP,ACTA2,COL1A1
Liver	Kupffer cells	CD68,MARCO,CD163,CSF1R,C1QA,C1QB,CLEC4F,TIMD4,VSIG4,ITGAM
Liver	Liver progenitor cell	EPCAM,KRT19,SOX9,PROM1,CD24,AFP,LGR5
Liver	Pit cells	PTPRC,CD3D,NCAM1,NKG7,KLRF1,FCGR3A,CXCR6,ITGA1,PRF1,GZMB
Liver	Endothelial cell	PECAM1,CDH5,STAB1,STAB2,LYVE1,VWF,KDR,ICAM1,CLEC4G,CLEC4M
Liver	Hematopoietic cell	PTPRC,CD34,KIT,RUNX1,SPN
Liver	Hematopoeitic precursor	CD34,CD38,CD133,CD117,CD90,CD48,CD150
Liver	Fibroblasts	PDGFRA,FBLN2,DCN,LUM,THY1,CD34,COL1A1,COL1A2,VIM
Liver	Mesenchymal stem cell	ENG,NT5E,THY1,PDGFRA,VIM,CD44,NES
Liver	Erythrocytes	HBB,HBA1,HBA2,GYPA,GYPB,SLC4A1,ALAS2,FECH,EPB41,SPTA1,CA1
Liver	Mast cells	KIT,FCER1A,CPA3,TPSAB1,TPSB2,MS4A2
Liver	Neutrophils	FCGR3B,CSF3R,MPO,ELANE,PRTN3,S100A8,S100A9,ITGAM,CEACAM8,CXCR1,CXCR2,SELL
Liver	Naive B cells	CD19,MS4A1,CD79A,IGHM,IGHD,CD27,SELL,IL4R,CCR7,BACH2
Liver	Memory B cells	CD19,MS4A1,CD79A,CD27,CD38,SDC1,IGHG1,IGHG2,IGHG3,IGHG4,IGHA1,IGHA2,AICDA,BANK1,PAX5
Liver	Plasma B cells	SDC1,CD38,XBP1,PRDM1,SLAMF7,DERL3,MZB1,IGHG1,IGHG2,IGHG3,IGHG4,IGHA1,IGHA2,IGLC2,IGKC
Liver	CD8+ NKT-like cells	CD3D,CD8A,CD8B,NCAM1,NKG7,KLRB1,KLRD1,GZMB,PRF1,TBX21,EOMES,SLAMF7
Liver	CD4+ NKT-like cells	CD3D,CD4,NCAM1,NKG7,KLRB1,GZMK,TBX21,EOMES,SLAMF6
Liver	Naive CD8+ T cells	CD3D,CD3E,CD3G,CD8A,CD8B,CCR7,SELL,LEF1,TCF7,IL7R,CD27,CD45RA
Liver	Naive CD4+ T cells	CD3D,CD3E,CD3G,CD4,CCR7,SELL,LEF1,TCF7,IL7R,CD27,CD45RA,FOXP1
Liver	Memory CD8+ T cells	CD3D,CD3E,CD3G,CD8A,CD8B,CD44,IL7R,CD69,EOMES,TBX21,IFNG,GZMK,GZMB,CD45RO

Tissue	Cell Type	Markers
Intestine	Eosinophils	CCR3,IL5RA,SIGLEC8,EPX,RNASE2,RNASE3,PRG2,CLC,HPGDS,PTGDR2
Intestine	Basophils	FCER1A,CD200R3,ENPP3,IL3RA,CD33,TPSAB1,TPSB2,HDC,GATA2
Intestine	Mast cells	KIT,FCER1A,CPA3,TPSAB1,TPSB2,MS4A2,ENPP3,CD63,CD9
Intestine	Naive B cells	CD19,MS4A1,CD79A,IGHM,IGHD,CD27,SELL,IL4R,CCR7,BACH2
Intestine	Memory B cells	CD19,MS4A1,CD79A,CD27,CD38,SDC1,IGHG1,IGHG2,IGHG3,IGHG4,IGHA1,IGHA2,AICDA,BANK1,PAX5
Intestine	Plasma B cells	SDC1,CD38,XBP1,PRDM1,SLAMF7,DERL3,MZB1,IGHG1,IGHG2,IGHG3,IGHG4,IGHA1,IGHA2,IGLC2,IGKC
Intestine	Naive CD8+ T cells	CD3D,CD3E,CD3G,CD8A,CD8B,CCR7,SELL,LEF1,TCF7,IL7R,CD27,CD45RA
Intestine	Naive CD4+ T cells	CD3D,CD3E,CD3G,CD4,CCR7,SELL,LEF1,TCF7,IL7R,CD27,CD45RA,FOXP1
Intestine	Memory CD8+ T cells	CD3D,CD3E,CD3G,CD8A,CD8B,CD44,IL7R,CD69,EOMES,TBX21,IFNG,GZMK,GZMB,CD45RO
Intestine	Memory CD4+ T cells	CD3D,CD3E,CD3G,CD4,CD44,IL7R,CD69,ICOS,CCR5,CCR6,CXCR3,TBX21,CD45RO
Intestine	Effector CD8+ T cells	CD3D,CD3E,CD3G,CD8A,CD8B,GZMB,PRF1,IFNG,KLRG1,CX3CR1,TBX21,EOMES,CCL5,CD27,SELL
Intestine	Effector CD4+ T cells	CD3D,CD3E,CD3G,CD4,IFNG,TNF,IL2,IL4,IL17A,IL21,ICOS,CD40LG,TBX21,GATA3,RORC,BCL6,PRDM1
Intestine	γδ-T cells	CD3D,CD3E,CD3G,TRDC,TRGC1,TRGC2,NKG7,KLRB1,KLRG1,CCL5,CD160
Intestine	CD8+ NKT-like cells	CD3D,CD8A,CD8B,NCAM1,NKG7,KLRB1,KLRD1,GZMB,PRF1,TBX21,EOMES,SLAMF7
Intestine	CD4+ NKT-like cells	CD3D,CD4,NCAM1,NKG7,KLRB1,GZMK,TBX21,EOMES,SLAMF6
Intestine	Natural killer cells	NCAM1,NKG7,KLRF1,KLRD1,KLRB1,KLRC1,KLRC2,FCGR3A,GZMB,PRF1,EOMES,TBX21
Intestine	Platelets	ITGA2B,ITGB3,GP1BA,GP9,PPBP,PF4,SELP,TUBB1,CD9
Intestine	Megakaryocyte	ITGA2B,ITGB3,GP1BA,GP9,PF4,PPBP,VWF,MPL,FLI1,GATA1,TUBB1
Intestine	Erythroid cells	HBB,HBA1,HBA2,HBG1,HBG2,GYPA,GYPB,ALAS2,FECH,SLC4A1,EPOR,GATA1,KLF1
Intestine	Erythroblasts	HBB,HBA1,HBA2,GYPA,ALAS2,GATA1,KLF1
Pancreas	Acinar cells	PRSS1,CPA1,CPA2,CELA3A,AMY2A,PNLIP,REG1A,CTRB1,CTRB2,KLK1
Pancreas	Alpha cells	GCG,ARX,TTR,IRX2,MAFB,GC
Pancreas	Beta cells	INS,IAPP,MAFA,PDX1,NKX6-1,SIX2,SIX3,PCSK1
Pancreas	Delta cells	SST,HHEX,LEPR,RBP4
Pancreas	Ductal cells	KRT19,KRT7,SOX9,CFTR,SPP1,S100P,FOXA2,HNF1B
Pancreas	Epsilon cells	GHRL
Pancreas	Gamma (PP) cells	PPY,PAX6
Pancreas	Pancreatic progenitor cells	PDX1,SOX9,NKX6-1,PTF1A,NEUROG3,HNF1B,GATA6
Pancreas	Pancreatic stellate cells	ACTA2,PDGFRB,RGS5,COL1A1,COL1A2,VIM,DES,DCN,LUM
Pancreas	Peri-islet Schwann cells	S100B,SOX10,PLP1,MPZ,CDH19
Pancreas	Endothelial cells	PECAM1,CDH5,VWF,ENG,PLVAP,KDR,FLT1
Pancreas	Mesenchymal cells	VIM,COL1A1,COL1A2,PDGFRA,PDGFRB,DCN,LUM
Pancreas	Mast cells	KIT,FCER1A,CPA3,TPSAB1,TPSB2,MS4A2
Pancreas	Immune system cells	PTPRC,CD3E,CD19,CD68,NCAM1,ITGAX,KIT
Pancreas	Cancer stem cells	EPCAM,PROM1,CD44,ALDH1A1,CD24,CXCR4,SOX2,POU5F1
Kidney	Proximal tubule cells	SLC5A2,SLC5A1,LRP2,CUBN,SLC3A1,SLC7A9,SLC22A6,SLC22A8,AQP1,ALDOB,HNF4A
Kidney	Loop of Henle cells	UMOD,SLC12A1,KCNJ1,CLCNKB,BSND
Kidney	Distal tubule cells	SLC12A3,TRPM6,KLK1,PVALB,WNK1
Kidney	Connecting tubule cells	CALB1,TRPV5,SLC8A1,KL,AQP2,SCNN1G
Kidney	Principal cells (Collecting duct system)	AQP2,AQP3,AQP4,FXYD4,HSD11B2,SCNN1A,SCNN1B,SCNN1G
Kidney	Inner Medullary cells (Collecting duct system)	AQP2,HSD11B2,SCNN1G,SLC14A2
Kidney	α-intercalated cells (Collecting duct system)	ATP6V1G3,ATP6V0D2,SLC4A1,FOXI1,INS RR
Kidney	β-intercalated cells (Collecting duct system)	SLC26A4,FOXI1,INS RR
Kidney	Podocytes	NPHS1,NPHS2,PODXL,WT1,SYNPO,VEGFA,MAFB,CD2AP
Kidney	Mesangial cells	PDGFRB,ACTA2,ITGA8,GATA3,MEIS1,REN
Kidney	Juxtaglomerular cells	REN,ACTA2,GJA5,CDH11,PDE3B,PVALB
Kidney	Endothelial cells	PECAM1,CDH5,EMCN,KDR,FLT1,VWF
Kidney	Stromal cells	PDGFRA,COL1A1,COL1A2,DCN,LUM,MEIS1,FOXD1
Kidney	Renal interstitium (Mesenchymal cells)	PDGFRA,COL1A1,DCN,LUM,FOXD1,CLDN1,VIM
Kidney	Cap mesenchyme cells (Mesenchymal cells)	SIX2,CITED1,EYA1,GDNF,OSR1
Kidney	Ureteric Bud cells	RET,WNT11,GFRA1,HOXB7,CALB1,KRT19,EPCAM
Kidney	Renal Vesicle cells	LHX1,PAX8,WT1,CDH6
Kidney	Hematopoietic cells	PTPRC,CD34,KIT,GYPA,HBB
Kidney	Immune cells	PTPRC,CD68,CD3E,CD19,ITGAM,FCGR3A,NCAM1
Kidney	Cancer stem cells	PROM1,CD44,ALDH1A1,EPCAM,SOX2,POU5F1,NANOG,ABCG2,CD133(PROM1)
Brain	Astrocytes	GFAP,AQP4,SLC1A2,SLC1A3,S100B,ALDH1L1,ALDOC,SOX9,FGFR3
Brain	Oligodendrocytes	MBP,PLP1,MOG,MAG,CNP,OLIG1,OLIG2,SOX10,NKX6-2,KLK6
Brain	Oligodendrocyte precursor cells	PDGFRA,CSPG4,SOX10,OLIG1,OLIG2,ASCL1,NKX2-2
Brain	Microglial cells	AIF1,CSF1R,CX3CR1,P2RY12,TMEM119,CD68,HEXB,C1QA,SPI1,IRF8
Brain	Glutamatergic neurons	SLC17A6,SLC17A7,SLC17A8,TBR1,SATB2,CUX1,CUX2,BCL11B,FEZF2,FOXP2
Brain	GABAergic neurons	GAD1,GAD2,SLC32A1,DLX1,DLX2,DLX5,ARX,LHX6,SST,NPY,VIP,CALB1,CALB2,PVALB
Brain	Dopaminergic neurons	TH,SLC6A3,SLC18A2,DDC,NR4A2,EN1,LMX1A,LMX1B,PITX3
Brain	Serotonergic neurons	TPH2,SLC6A4,FEV,GATA3,LMX1B,PET1(FEV)
Brain	Cholinergic neurons	CHAT,SLC5A7,SLC18A3,ACHE,NGFR
Brain	Mature neurons	RBFOX3,TUBB3,MAP2,SYP,SYN1,SNAP25,NEFL,NEFM,NEFH
Brain	Immature neurons	DCX,TUBB3,STMN2,NEUROD1,NEUROD2,NEUROG1,NEUROG2,ELAVL4
Brain	Neuroblasts	DCX,TUBB3,STMN2,NEUROD1,TAGLN3,ASCL1
Brain	Neural Progenitor cells	NES,SOX2,PAX6,OLIG2,ASCL1,MSI1,FABP7,HES1,HES5
Brain	Neural stem cells	NES,SOX2,PAX6,PROM1,GFAP,ID1,MSI1,FABP7,HOPX
Brain	Neuroepithelial cells	SOX2,PAX6,NES,HES1,HES5,CDH2
Brain	Radial glial cells	GFAP,VIM,NES,SOX2,PAX6,FABP7,SLC1A3,HES1,HES5
Brain	Ependymal Cells	FOXJ1,RSPH1,PIFO,CD24,GFAP,S100B,VIM,TMEM212
Brain	Choroid Plexus Epithelial Cells	TTR,AQP1,KRT8,KRT18,OTX2,ENPP2,CLIC6,FOLR1,CDH1
Brain	Tanycytes	RAX,COL25A1,DIO2,SLC16A2,GFAP,VIM,PPP1R1B
Brain	Myelinating Schwann cells	MPZ,MBP,PMP22,MAG,PLP1,SOX10,EGR2,POU3F1
Brain	Non myelinating Schwann cells	S100B,SOX10,NCAM1,CDH19,GFAP,NGFR
Brain	Schwann precursor cells	SOX10,FABP7,MPZ,PLP1,ERBB3
Brain	Endothelial cells	PECAM1,CDH5,CLDN5,VWF,SLC2A1,ABCB1,ICAM1,PLVAP
Brain	Immune system cells	PTPRC,AIF1,CD68,CSF1R,CX3CR1,P2RY12,TMEM119,CD3E,MS4A1
Brain	Cancer cells	GFAP,OLIG2,EGFR,PDGFRA,IDH1,IDH2,ATRX,MKI67,SOX2,NES,MYC,OTX2
Brain	Cancer stem cells	PROM1,CD44,NES,SOX2,OLIG2,L1CAM,ALDH1A1,MSI1
Eye	Rod photoreceptor cells	RHO,SAG,PDE6B,PDE6A,GNGT1,ROM1,PRPH2,NRL,NR2E3,RCVRN
Eye	Cone photoreceptor cells	OPN1SW,OPN1MW,OPN1LW,ARR3,PDE6C,PDE6H,GNGT2,RXRG,THRB,RCVRN
Eye	Retinal ganglion cells	POU4F1,POU4F2,THY1,NEFL,NEFM,SNCG,GAP43,TUBB3,RBPMS,SLC17A6
Eye	Rod bipolar cells	PRKCA,CABP5,TRPM1,GRIK1,LRIT3,PCP2
Eye	Cone bipolar cells	PRKCA,CABP5,GRIK1,ISL1,GRM6,VSX1,VSX2,NKX2-2
Eye	Horizontal cells	CALB1,LHX1,PROX1,PCP4,ONECUT1,ONECUT2
Eye	GABAergic amacrine cells	GAD1,GAD2,SLC32A1,PAX6,CALB1,CALB2,NPY,SOM,VIP
Eye	Glycinergic amacrine cells	SLC6A9,PAX6,SLC32A1,EBF3
Eye	Cholinergic GABAergic amacrine cells	CHAT,SLC5A7,SLC18A3,GAD1,GAD2,SLC32A1
Eye	Tachykinin GABAergic amacrine cells	TAC1,GAD1,GAD2,SLC32A1
Eye	Starburst amacrine cells	CHAT,SLC18A3,SLC5A7,FEZF1,GAD1,GAD2,SLC32A1
Eye	Muller cells	RLBP1,GLUL,SLC1A3,AQP4,VIM,CD44,SOX2,PAX6
Eye	Retinal pigment epithelial cells	RPE65,BEST1,RLBP1,PMEL,TYR,DCT,MITF,KRT8,KRT18,TRPM1
Eye	Retinal progenitor cells	PAX6,SOX2,VSX2,LHX2,NES,CCND1,ASCL1,OLIG2

Tissue	Cell Type	Markers	Tissue	Cell Type	Markers
Liver	Memory CD4+ T cells	CD3D,CD3E,CD3G,CD4,CD44,IL7R,CD69,ICOS,CCR5,CCR6,CXCR3,TBX21,GATA3,RORC,FOXP3,CD45RO	Eye	Cornea epithelial cells	KRT12,KRT3,KRT5,KRT14,TP63,PAX6,EPCAM
Liver	Effector CD8+ T cells	CD3D,CD3E,CD3G,CD8A,CD8B,GZMB,PRF1,IFNG,KLRG1,CX3CR1,TBX21,EOMES,CCL5,CD27,SELL	Eye	Corneal Endothelial Cells	SLC4A11,AQP1,PITX2,COL8A2,CDH2,ATP1A1,TJP1
Liver	Effector CD4+ T cells	CD3D,CD3E,CD3G,CD4,IFNG,TNF,IL2,IL4,IL17A,IL21,ICOS,CD40LG,TBX21,GATA3,RORC,BCL6,PRDM1	Eye	Lens Epithelial Cells	CRYAA,CRYAB,PAX6,FOXE3,KRT19,HSF4,CDH1,LIMS1
Liver	T regulatory cells	FOXP3,IL2RA,CTLA4,IKZF2,TNFRSF18,CD4,PTPRC,LAG3	Eye	Astrocytes	GFAP,AQP4,SLC1A2,S100B,ALDH1L1,ALDOC
Liver	γδ-T cells	CD3D,CD3E,CD3G,TRDC,TRGC1,TRGC2,NKG7,KLRB1,KLRG1,CCL5,CD160	Eye	Microglial cells	AIF1,CD68,CSF1R,CX3CR1,P2RY12,TMEM119,HEXB,C1QA
Liver	Classical Monocytes	CD14,FCGR3A,CCR2,S100A8,S100A9,LYZ,VCAN,FCN1,CD33	Eye	Endothelial cells	PECAM1,CDH5,VWF,ICAM1,CLDN5,PLVAP
Liver	Non-classical monocytes	FCGR3A,CD14,CX3CR1,CSF1R,ITGAL,LST1,MS4A7,SIGLEC10,TCF7L2	Eye	Pericytes	PDGFRB,RGS5,ACTA2,MCAM,CSPG4,KCNJ8,ABCC9,CD146
Liver	Intermediate monocytes	CD14,FCGR3A,CCR2,HLA-DRA,CD86,CCR5,ITGAX,CLEC10A	Eye	Fibroblasts	COL1A1,COL1A2,VIM,DCN,LUM,PDGFRA,FN1
Liver	Myeloid Dendritic cells	ITGAX,HLA-DRA,HLA-DRB1,HLA-DQA1,CD1C,FCER1A,CLEC10A,CD83,CD86,CCR7,IRF4,IRF8,SIRPA,CLEC9A	Eye	Immune cells	PTPRC,AIF1,CD68,CD3E,CD19,ITGAM
Liver	Plasmacytoid Dendritic cells	IL3RA,CLEC4C,NRP1,TCF4,IRF7,IRF8,LILRA4,GZMB,SPIB,CD123	Eye	Synapses	SYP,SYN1,SNAP25,STX1A,SYT1,CADM1,NLGN1,NRXN1
Lung	Cancer stem cells	EPCAM,PROM1,CD44,ALDH1A1,CD24,THY1,NANOG,SOX2	Eye	Extracellular matrix cells	COL1A1,COL4A1,FN1,LAMB1,VCAN,DCN,LUM,ELN
Lung	Pulmonary alveolar type I cells	AGER,PDPN,CAV1,ICAM1,CLIC5,HOPX,AQP5	Eye	Cancer cells	PMEL,MLANA,SOX10,MITF,TYR,MKI67,RB1,CRX,ARR3
Lung	Pulmonary alveolar type II cells	SFTPC,SFTPB,SFTPA1,SFTPA2,SFTPD,LAMP3,ABCA3,ETV5,SOX9,NAPSA	Heart	Cardiomyocytes	TNNT2,TNNC1,TNNI3,MYH6,MYH7,ACTC1,NPPA,NPPB,MYL2,MYL7,NKX2-5,GATA4,TBX5
Lung	Clara cells	SCGB1A1,SCGB3A2,CYP2F1,UPK3A,CEACAM6	Heart	Cardiac Conduction System Cells	HCN4,TBX3,TBX5,SHOX2,ISL1,SCN5A,CACNA1H,GJA5,KCND2,NKX2-5
Lung	Basal cells	KRT5,KRT14,KRT15,TP63,NGFR,SOX2,NOTCH1	Heart	Purkinje Fibers	GJA5,SCN5A,KCND2,ID2,RYR2,MYL2,ATP2A2,TNNT2,ACTC1
Lung	Ciliated cells	FOXJ1,PIFO,DYNC2LI1,DNAH5,CCDC39,RSPH1,TP73	Heart	Endocardial cells	NPR3,CDH5,PECAM1,NFATC1,HEY1,MSX1
Lung	Lung multiciliated epithelial cell	FOXJ1,TPPP3,CADPS,PIFO,DNAH5	Heart	Vascular endothelial cells	PECAM1,CDH5,VWF,ICAM1,ENG,KDR,FLT1
Lung	Ionocytes	FOXI1,CFTR,ASCL3,ATP6V0D2,STAP1	Heart	Lymphatic endothelial cells	PROX1,LYVE1,PDPN,FLT4,CCL21,PECAM1,CDH5
Lung	Airway goblet cells	MUC5AC,MUC5B,SPDEF,FOXA3,TFF1,TFF2,TFF3,REG4	Heart	Stromal cells	PDGFRA,PDGFRB,DCN,LUM,COL1A1,COL1A2,VIM,TCF21,POSTN
Lung	Goblet / Secretory cell	SCGB1A1,MUC5AC,MUC5B,SCGB3A1,SCGB3A2,UPK3A	Heart	Smooth muscle cells	ACTA2,TAGLN,MYH11,CNN1,DES,CALD1
Lung	Bronchus Serous cell	LPO,BPIFB1,LTF,PRR4,SLPI,BPIFA1	Heart	Epicardial fat cells	ADIPOQ,PLIN1,LEP,FABP4,LIPE,CEBPA,PPARG
Lung	Epithelial cells	EPCAM,KRT8,KRT18,CDH1	Heart	Schwann cells	S100B,SOX10,PLP1,MPZ,GFAP
Lung	Pulmonary Neuroendocrine Cells	ASCL1,CHGA,CHGB,SYP,GRP,CALCA,UCHL1,INSM1,EPCAM	Heart	Visceral neurons	TH,CHAT,NOS1,TUBB3,ELAVL4,PHOX2B,NGF
Lung	Mesothelial cells	MSLN,WT1,UPK3B,CALB2,LRRN4	Heart	Myeloid cells	PTPRC,CD68,MRC1,CD163,ITGAM,CSF1R,ADGRE1,CCR2
Lung	Mesothelial cells	MSLN,WT1,UPK3B,CALB2,KRT8,KRT18,CDH1	Heart	Lymphoid cells	PTPRC,CD3E,CD8A,CD4,CD19,MS4A1,NCAM1,CCR7,IL7R
Lung	Endothelial cell	PECAM1,CDH5,VWF,ICAM1,EMCN,ACKR1,PLVAP,CA4	Heart	Megakaryocytes	ITGA2B,ITGB3,GP1BA,PF4,PPBP
Lung	Capillary endothelial cells	EDNRB,CA4,CD36,SGK1,RGCC	Heart	Erythroblasts	HBB,HBA1,HBA2,GYPA,ALAS2,GATA1,KLF1
Lung	Capillary endothelial cell	PECAM1,CA4,RGCC,SGK1,PRX	Heart	SATB2_LRRC7 positive cells	SATB2,LRRC7
Lung	Bronchial endothelial cells	VWF,ACKR1,SELE,BMPER	Heart	ELF3_AGBL2 positive cells	ELF3,AGBL2
Lung	Fibroblasts	COL1A1,COL1A2,COL3A1,PDGFRA,PDGFRB,DCN,LUM,VIM,FN1,ACTA2	Heart	CLC_IL5RA positive cells	CLC,IL5RA
Lung	Adventitial fibroblasts	PI16,MFAP5,SCARA5,DCN,LUM	Adrenal	Adrenocortical cells	CYP11B1,CYP11B2,CYP17A1,CYP21A2,HSD3B2,MC2R,NR5A1,STAR
Lung	Pericytes	COX4I2,PDGFRB,RGS5,CSPG4,KCNJ8	Adrenal	Chromaffin cells	CHGA,CHGB,TH,DBH,PNMT,SYP,SLC18A1
Lung	Vascular associated smooth muscle cell	ACTA2,MYH11,NOTCH3,CNN1,RGS5	Adrenal	Sympathoblasts	TH,PHOX2B,DBH,ASCL1,SOX10,CHGA
Lung	Bronchial smooth muscle cell	ACTA2,MYH11,HHIP,DES,CALD1	Adrenal	Schwann cells	S100B,SOX10,PLP1,MPZ,CDH19
Lung	Alveolar macrophages	CD68,MRC1,MARCO,MSR1,FABP4,PPARG,SIGLEC1,SIGLECF	Adrenal	Stromal cells	COL1A1,COL1A2,VIM,DCN,PDGFRA,PDGFRB
Lung	Macrophages	CD68,CD163,MRC1,CSF1R,CD14,ITGAM,ADGRE1,APOE,C1QA,C1QB,C1QC,MARCO,MSR1	Adrenal	Vascular endothelial cells	PECAM1,CDH5,VWF,ENG,PLVAP
Lung	Classical Monocytes	CD14,FCGR3A,CCR2,S100A8,S100A9,LYZ,VCAN,FCN1,CD33	Adrenal	Myeloid cells	PTPRC,CD68,ITGAM,ADGRE1,CSF1R,CD14,FCGR3A
Lung	Non-classical monocytes	FCGR3A,CD14,CX3CR1,CSF1R,ITGAL,LST1,MS4A7,SIGLEC10,TCF7L2	Adrenal	Lymphoid cells	PTPRC,CD3E,CD8A,CD4,CD19,MS4A1,NCAM1
Lung	Intermediate monocytes	CD14,FCGR3A,CCR2,HLA-DRA,CD86,CCR5,ITGAX,CLEC10A	Adrenal	Megakaryocytes	ITGA2B,ITGB3,GP1BA,PF4,PPBP
Lung	Myeloid Dendritic cells	ITGAX,HLA-DRA,HLA-DRB1,HLA-DQA1,CD1C,FCER1A,CLEC10A,CD83,CD86,CCR7,IRF4,IRF8,SIRPA,CLEC9A	Adrenal	Erythroblasts	HBB,HBA1,HBA2,GYPA,ALAS2,GATA1,KLF1
Lung	Plasmacytoid Dendritic cells	IL3RA,CLEC4C,NRP1,TCF4,IRF7,IRF8,LILRA4,GZMB,SPIB,CD123	Adrenal	CSH1_CSH2 positive cells	CSH1,CSH2
Lung	Granulocytes	MPO,ELANE,PRTN3,AZU1,S100A8,S100A9,CEACAM8,FCGR3B,CSF3R	Adrenal	SLC26A4_PAEP positive cells	SLC26A4,PAEP
Lung	Neutrophils	FCGR3B,CSF3R,MPO,ELANE,PRTN3,S100A8,S100A9,ITGAM,CEACAM8,CXCR1,CXCR2	Muscle	Skeletal muscle cells	MYH1,MYH2,MYH4,MYH7,ACTN2,ACTN3,TNNT1,TNNT3,CKM,DES,MYOD1,MYOG
Lung	Eosinophils	CCR3,IL5RA,SIGLEC8,EPX,RNASE2,RNASE3,PRG2,CLC,HPGDS,PTGDR2	Muscle	Satellite cells	PAX7,MYF5,CD34,CAV1,VCAM1,NCAM1,ITGA7
Lung	Basophils	FCER1A,CD200R3,ENPP3,IL3RA,CD33,TPSAB1,TPSB2,HDC,GATA2	Muscle	Fibro-adipogenic Progenitors	PDGFRA,LY6A,VIM,COL1A1,DCN,CD34,PPARG,CEBPA
Lung	Mast cells	KIT,FCER1A,CPA3,TPSAB1,TPSB2,MS4A2,ENPP3,CD63,CD9	Muscle	Smooth muscle cells	ACTA2,TAGLN,MYH11,CNN1,DES
Lung	Naive B cells	CD19,MS4A1,CD79A,IGHM,IGHD,CD27,SELL,IL4R,CCR7,BACH2	Muscle	Stromal cells	PDGFRA,COL1A1,VIM,DCN,LUM,TCF4,ASPN
Lung	Memory B cells	CD19,MS4A1,CD79A,CD27,CD38,SDC1,IGHG1,IGHG2,IGHG3,IGHG4,IGHA1,IGHA2,AICDA,BANK1,PAX5	Muscle	Vascular endothelial cells	PECAM1,CDH5,VWF,ENG,KDR,ICAM1
Lung	Plasma B cells	SDC1,CD38,XBP1,PRDM1,SLAMF7,DERL3,MZB1,IGHG1,IGHG2,IGHG3,IGHG4,IGHA1,IGHA2,IGLC2,IGKC	Muscle	Lymphatic endothelial cells	PROX1,LYVE1,PDPN,FLT4,PECAM1,CDH5
Lung	Naive CD8+ T cells	CD3D,CD3E,CD3G,CD8A,CD8B,CCR7,SELL,LEF1,TCF7,IL7R,CD27,CD45RA	Muscle	Schwann cells	S100B,SOX10,PLP1,MPZ,GFAP,NGFR
Lung	Naive CD4+ T cells	CD3D,CD3E,CD3G,CD4,CCR7,SELL,LEF1,TCF7,IL7R,CD27,CD45RA,FOXP1	Muscle	Myeloid cells	PTPRC,CD68,MRC1,ITGAM,ADGRE1,CSF1R
Lung	Memory CD8+ T cells	CD3D,CD3E,CD3G,CD8A,CD8B,CD44,IL7R,CD69,EOMES,TBX21,IFNG,GZMK,GZMB,CD45RO	Muscle	Lymphoid cells	PTPRC,CD3E,CD4,CD8A,NCAM1
Lung	Memory CD4+ T cells	CD3D,CD3E,CD3G,CD4,CD44,IL7R,CD69,ICOS,CCR5,CCR6,CXCR3,TBX21,GATA3,RORC,FOXP3,CD45RO	Muscle	Megakaryocytes	ITGA2B,ITGB3,GP1BA,PF4,PPBP
Lung	Effector CD8+ T cells	CD3D,CD3E,CD3G,CD8A,CD8B,GZMB,PRF1,IFNG,KLRG1,CX3CR1,TBX21,EOMES,CCL5,CD27,SELL	Muscle	Erythroblasts	HBB,HBA1,HBA2,GYPA,ALAS2,GATA1,KLF1
Lung	Effector CD4+ T cells	CD3D,CD3E,CD3G,CD4,IFNG,TNF,IL2,IL4,IL17A,IL21,ICOS,CD40LG,TBX21,GATA3,RORC,BCL6,PRDM1	Stomach	Parietal and chief cells	ATP4A,ATP4B,GIF,PGA3,PGA4,PGC,LIPF,BHLHA15
Lung	gamma delta-T cells	CD3D,CD3E,CD3G,TRDC,TRGC1,TRGC2,NKG7,KLRB1,KLRG1,CCL5,CD160	Stomach	Neuroendocrine cells	CHGA,CHGB,SYP,NCAM1,SST,GAST,GHRL,TPH1,INS L1
Lung	CD8+ NKT-like cells	CD3D,CD8A,CD8B,NCAM1,NKG7,KLRB1,KLRD1,GZMB,PRF1,TBX21,EOMES,SLAMF7	Stomach	Goblet cells	MUC2,TFF3,FCGBP,SPDEF
Lung	CD4+ NKT-like cells	CD3D,CD4,NCAM1,NKG7,KLRB1,GZMK,TBX21,EOMES,SLAMF6	Stomach	Ciliated epithelial cells	FOXJ1,PIFO,KRT8,KRT18,EPCAM
Lung	Natural killer cells	NCAM1,NKG7,KLRF1,KLRD1,KLRB1,KLRC1,KLRC2,FCGR3A,GZMB,PRF1,EOMES,TBX21	Stomach	Squamous epithelial cells	KRT5,KRT14,TP63
Lung	Platelets	ITGA2B,ITGB3,GP1BA,GP9,PPBP,PF4,SELP,TUBB1,CD9	Stomach	Stromal cells	COL1A1,COL1A2,VIM,DCN,LUM,PDGFRA,PDGFRB,POSTN
Lung	Megakaryocyte	ITGA2B,ITGB3,GP1BA,GP9,PF4,PPBP,VWF,MPL,FLI1,GATA1,TUBB1	Stomach	Vascular endothelial cells	PECAM1,CDH5,VWF,ENG,PLVAP
Lung	Erythroid cells	HBB,HBA1,HBA2,HBG1,HBG2,GYPA,GYPB,ALAS2,FECH,SLC4A1,EPOR,GATA1,KLF1	Stomach	Lymphatic endothelial cells	PROX1,LYVE1,PDPN,FLT4
Lung	Progenitor cells	CD34,KIT,FLT3,MPO,AZU1,PRTN3,ELANE,GATA1,GATA2,MYB	Stomach	Mesothelial cells	MSLN,WT1,UPK3B,CALB2
Intestine	Cancer stem cells	EPCAM,PROM1,CD44,ALDH1A1,SOX2,POU5F1,NANOG,KRT5,TP63	Stomach	ENS neurons	TUBB3,ELAVL4,PHOX2B,CHAT,NOS1,VIP,GAL
Intestine	Enterocytes	FABP2,ALPI,VIL1,SI,SLC26A3,ANPEP,KRT20,FABP1,SLC5A1,ACE2	Stomach	ENS glia	S100B,SOX10,PLP1,FABP7,GFAP
Intestine	Intestinal epithelial cells	EPCAM,CDH1,KRT8,KRT18,VIL1	Stomach	Myeloid cells	PTPRC,CD68,MRC1,ITGAM,ADGRE1,CD14,KIT,FCER1A
Intestine	Intestinal Stem Cells	LGR5,OLFM4,ASCL2,SOX9,SMOC2,RGMB,BMI1,TERT,EPCAM	Stomach	Lymphoid cells	PTPRC,CD3E,CD4,CD8A,CD19,MS4A1,NCAM1,ITGAE
Intestine	Transit amplifying cells	MKI67,TOP2A,PCNA,OLFM4,EPHB2,SOX9,STMN1,MCM5,CENPF	Stomach	Erythroblasts	HBB,HBA1,HBA2,GYPA,ALAS2
Intestine	Crypt cells	LGR5,ASCL2,OLFM4,SOX9,SMOC2,EPHB2,CD44,AXIN2,RGMB	Stomach	MUC13_DMBT1 positive cells	MUC13,DMBT1
Intestine	Goblet cells	MUC2,TFF3,FCGBP,ZG16,CLCA1,AGR2,SPDEF	Stomach	PDE1C_ACSM3 positive cells	PDE1C,ACSM3
Intestine	Paneth Cells	LYZ,DEFA5,DEFA6,MMP7,PLA2G2A,REG3A,ITLN2	Spleen	Lymphoid cells	PTPRC,CD3E,CD4,CD8A,CD19,MS4A1,CR2,NCAM1,IGHM,IGHD,AICDA,FOXP3
Intestine	Tuft Cells	POU2F3,TRPM5,IL25,AVIL,DCLK1,GFI1B,HCK,EPCAM	Spleen	Myeloid cells	PTPRC,CD68,CD163,MRC1,ITGAM,ADGRE1,CD14,ITGAX,CLEC4C,FCER1A
Intestine	Tuft cells	DCLK1,POU2F3,TRPM5,SH2D6,BMX,AVIL,LRMP,GFI1B,HCK,IL25	Spleen	Stromal cells	PDGFRA,PDGFRB,COL1A1,VIM,DCN,LUM,CCL19,CCL21,MADCAM1,LTBR,CR2
Intestine	M cells	SPIB,GP2,TNFRSF11A,CCL9,CKLF,MARCKSL1,ANXA5,EPCAM	Spleen	Vascular endothelial cells	PECAM1,CDH5,VWF,ENG,ACKR1,SELP
Intestine	Enteroendocrine cells	CHGA,CHGB,SYP,NEUROD1,TPH1,GCG,PYY,CCK,SCT,GIP,SST	Spleen	Mesothelial cells	MSLN,WT1,UPK3B,CALB2
Intestine	Chromaffin cells	CHGA,TH,TPH1,DDC	Spleen	Megakaryocytes	ITGA2B,ITGB3,GP1BA,PF4,PPBP,MPL
Intestine	Enteroglial cells	S100B,GFAP,SOX10,PLP1,CDH19,FOXD3,GPM6B,CRYAB,ERBB3	Spleen	Erythroblasts	HBB,HBA1,HBA2,GYPA,ALAS2,GATA1,KLF1
Intestine	ENS glia	S100B,SOX10,PLP1,FABP7,GFAP,BFABP(FABP7)	Spleen	AFP_ALB positive cells	AFP,ALB
Intestine	Fibroblasts	COL1A1,COL1A2,COL3A1,PDGFRA,DCN,LUM,FN1,ACTA2	Spleen	STC2_TLX1 positive cells	STC2,TLX1
Intestine	Myofibroblast cells	COL1A1,POSTN,TIMP1,ACTA2,TAGLN,FN1,CNN1	Thymus	Thymocytes	PTPRC,IL7R,CD3D,CD3E,CD4,CD8A,CD8B,CD1A,CD44,CD24,CD69,RAG1,RAG2,PTCRA,TRBC1,TRBC2,FOXP3,IKZF1
Intestine	Smooth muscle cells	ACTA2,TAGLN,MYH11,CNN1,CALD1,SMTN	Thymus	Thymic epithelial cells	EPCAM,KRT8,KRT5,KRT14,CDH1,FOXN1,AIRE,CCL25,PSMB11,CD83,CD40,HLA-DRA
Intestine	Pericytes	RGS5,NOTCH3,MCAM,KCNJ8,ABCC9,HIGD1B,GJA4	Thymus	Antigen presenting cells	HLA-DRA,HLA-DRB1,CD80,CD86,CD40,ITGAX,CD68,CLEC4C,IL3RA
Intestine	Vascular endothelial cells	PECAM1,CDH5,VWF,ENG,PLVAP,SELE	Thymus	Stromal cells	PDGFRA,COL1A1,VIM,DCN,LUM,PDPN
Intestine	Lymphatic endothelial cells	PROX1,LYVE1,PDPN,FLT4,CCL21,PECAM1,CDH5	Thymus	Vascular endothelial cells	PECAM1,CDH5,VWF,ENG,SELE,ICAM1
Intestine	Mesothelial cells	MSLN,WT1,UPK3B,CALB2,KRT8,KRT18	Placenta	Syncytiotrophoblasts and villous cytotrophoblasts	KRT7,GJA1,CDH1,EPCAM,CGA,CGB,CYP19A1,ERVFRD-1,GCM1,TEAD4
Intestine	Innate Lymphoid Cell 1	PTPRC,ID2,IL7R,TBX21,IFNG,NKG7,NCR1,EOMES	Placenta	Extravillous trophoblasts	HLA-G,ITGA5,ITGA1,CDH1,KRT7,MMP2,MMP9,PAPPA2
Intestine	Innate Lymphoid Cell 2	PTPRC,ID2,IL7R,GATA3,IL5,IL13,IL1RL1,PTGDR2,ARG1	Placenta	Trophoblast giant cells	HAND1,ASCL2,GCM1,KRT7,CDH1
Intestine	Innate Lymphoid Cell 3	PTPRC,ID2,IL7R,RORC,IL22,IL17A,KIT,CCR6,NCR1	Placenta	Stromal cells	COL1A1,COL1A2,VIM,PDGFRA,PDGFRB,DCN,LUM
Intestine	Myeloid cells	PTPRC,CD68,MRC1,ITGAM,CSF1R,ADGRE1,CD14,FCGR3A,ITGAX,KIT	Placenta	Vascular endothelial cells	PECAM1,CDH5,VWF,ENG,KDR
Intestine	Macrophages	CD68,CD163,MRC1,CSF1R,CD14,ITGAM,ADGRE1,APOE,C1QA,C1QB,C1QC,MARCO,MSR1	Placenta	Smooth muscle cells	ACTA2,TAGLN,MYH11,CNN1
Intestine	Classical Monocytes	CD14,FCGR3A,CCR2,S100A8,S100A9,LYZ,VCAN,FCN1,CD33	Placenta	Myeloid cells	PTPRC,CD68,MRC1,CD163,CSF1R,ITGAM,CD14,LYZ
Intestine	Non-classical monocytes	FCGR3A,CD14,CX3CR1,CSF1R,ITGAL,LST1,MS4A7,SIGLEC10,TCF7L2	Placenta	Lymphoid cells	PTPRC,CD3E,CD8A,CD4,NCAM1,TRDC
Intestine	Intermediate monocytes	CD14,FCGR3A,CCR2,HLA-DRA,CD86,CCR5,ITGAX,CLEC10A	Placenta	Megakaryocytes	ITGA2B,ITGB3,GP1BA,PF4,PPBP
Intestine	Myeloid Dendritic cells	ITGAX,HLA-DRA,HLA-DRB1,HLA-DQA1,CD1C,FCER1A,CLEC10A,CD83,CD86,CCR7,IRF4,IRF8,SIRPA,CLEC9A	Placenta	PAEP_MECOM positive cells	PAEP,MECOM
Intestine	Plasmacytoid Dendritic cells	IL3RA,CLEC4C,NRP1,TCF4,IRF7,IRF8,LILRA4,GZMB,SPIB,CD123	Placenta	AFP_ALB positive cells	AFP,ALB
Intestine	Neutrophils	FCGR3B,CSF3R,MPO,ELANE,PRTN3,S100A8,S100A9,ITGAM,CEACAM8,CXCR1,CXCR2	Placenta	IGFBP1_DKK1 positive cells	IGFBP1,DKK1

Supplementary Table 3. Summary statistics comparing max mean annotation accuracy of CellTypeAI:model combinations (and Google's Gemini 2.5 Pro using CellTypeAI's engineered prompt) versus conventional annotation methods (CellTypist and ScType). n = 3. Where both groups' standard deviation (s.d.) = 0, means were directly compared (p set to 0.0 for unequal means). Where one group's s.d. = 0, a one-sample t-test was used. Where both groups' s.d. >0, a two-tailed Welch's t-test was used. $P < 0.05$ was considered significant. ns = not significant | * $p < 0.05$ | ** $p < 0.01$ | *** $p < 0.001$.

Comparison	Dataset	t-stat	p-value	Significance
CellTypeAI: Qwen3:235b vs CellTypist	PBMC3K	6.386937353	0.023647937	*
CellTypeAI: Qwen3:235b vs ScType	PBMC3K	10.87944414	0.008343052	**
CellTypeAI: Qwen3:32b vs CellTypist	PBMC3K	inf	0	***
CellTypeAI: Qwen3:32b vs ScType	PBMC3K	inf	0	***
CellTypeAI: Phi4:14b vs CellTypist	PBMC3K	0	1	ns
CellTypeAI: Phi4:14b vs ScType	PBMC3K	inf	0	***
Gemini 2.5 Pro vs CellTypist	PBMC3K	11.40266782	0.007603468	**
Gemini 2.5 Pro vs ScType	PBMC3K	23.3826859	0.001823987	**
CellTypeAI: Qwen3:235b vs CellTypist	StrokePBMC	1.010362971	0.418681641	ns
CellTypeAI: Qwen3:235b vs ScType	StrokePBMC	7.000372014	0.019801898	*
CellTypeAI: Qwen3:32b vs CellTypist	StrokePBMC	-2.020725942	0.18071197	ns
CellTypeAI: Qwen3:32b vs ScType	StrokePBMC	9.959292144	0.009931966	**
CellTypeAI: Phi4:14b vs CellTypist	StrokePBMC	1.732050808	0.225403331	ns
CellTypeAI: Phi4:14b vs ScType	StrokePBMC	8.577775428	0.013320035	*
Gemini 2.5 Pro vs CellTypist	StrokePBMC	-0.757772228	0.527702159	ns
Gemini 2.5 Pro vs ScType	StrokePBMC	3.734734554	0.064802518	ns
CellTypeAI: Qwen3:235b vs CellTypist	TS: Liver	inf	0	***
CellTypeAI: Qwen3:235b vs ScType	TS: Liver	inf	0	***
CellTypeAI: Qwen3:32b vs CellTypist	TS: Liver	inf	0	***
CellTypeAI: Qwen3:32b vs ScType	TS: Liver	inf	0	***
CellTypeAI: Phi4:14b vs CellTypist	TS: Liver	6.423021745	0.023392201	*
CellTypeAI: Phi4:14b vs ScType	TS: Liver	24.24871131	0.001696354	**
Gemini 2.5 Pro vs CellTypist	TS: Liver	18.35973856	0.002953518	**
Gemini 2.5 Pro vs ScType	TS: Liver	46.88084186	0.000454687	***
CellTypeAI: Qwen3:235b vs CellTypist	TS: Lung	25.78831202	0.001500293	**
CellTypeAI: Qwen3:235b vs ScType	TS: Lung	74.8630849	0.000178381	***
CellTypeAI: Qwen3:32b vs CellTypist	TS: Lung	13.38402897	0.005536153	**
CellTypeAI: Qwen3:32b vs ScType	TS: Lung	53.53611587	0.000348722	***
CellTypeAI: Phi4:14b vs CellTypist	TS: Lung	8.187876545	0.014590521	*
CellTypeAI: Phi4:14b vs ScType	TS: Lung	48.33996345	0.00042767	***
Gemini 2.5 Pro vs CellTypist	TS: Lung	7.072540798	0.01941147	*
Gemini 2.5 Pro vs ScType	TS: Lung	25.47558063	0.001537268	**
CellTypeAI: Qwen3:235b vs CellTypist	TS: Small Intestine	inf	0	***
CellTypeAI: Qwen3:235b vs ScType	TS: Small Intestine	inf	0	***
CellTypeAI: Qwen3:32b vs CellTypist	TS: Small Intestine	8.833459119	0.012574372	*
CellTypeAI: Qwen3:32b vs ScType	TS: Small Intestine	34.29460599	0.000849171	***
CellTypeAI: Phi4:14b vs CellTypist	TS: Small Intestine	7.732369677	0.016317093	*
CellTypeAI: Phi4:14b vs ScType	TS: Small Intestine	25.91890316	0.001485246	**
Gemini 2.5 Pro vs CellTypist	TS: Small Intestine	15.99599863	0.003885442	**
Gemini 2.5 Pro vs ScType	TS: Small Intestine	45.95028907	0.000473277	***
